# A 3D deep learning model to predict the diagnosis of dementia with Lewy bodies, Alzheimer’s disease, and mild cognitive impairment using brain 18F-FDG PET

**DOI:** 10.1007/s00259-021-05483-0

**Published:** 2021-07-30

**Authors:** Kobra Etminani, Amira Soliman, Anette Davidsson, Jose R. Chang, Begoña Martínez-Sanchis, Stefan Byttner, Valle Camacho, Matteo Bauckneht, Roxana Stegeran, Marcus Ressner, Marc Agudelo-Cifuentes, Andrea Chincarini, Matthias Brendel, Axel Rominger, Rose Bruffaerts, Rik Vandenberghe, Milica G. Kramberger, Maja Trost, Nicolas Nicastro, Giovanni B. Frisoni, Afina W. Lemstra, Bart N. M. van Berckel, Andrea Pilotto, Alessandro Padovani, Silvia Morbelli, Dag Aarsland, Flavio Nobili, Valentina Garibotto, Miguel Ochoa-Figueroa

**Affiliations:** 1 Center for Applied Intelligent Systems Research (CAISR), Halmstad University, Halmstad, Sweden; 2 Department of Clinical Physiology, Department of Health, Medicine and Caring Sciences, Linköping University, Linköping, Sweden; 3 National Cheng Kung University in Tainan, Tainan, Taiwan; 4 Department of Nuclear Medicine, Medical Imaging Area, Hospital Universitari i Politècnic La Fe, Valencia, Spain; 5 Servicio de Medicina Nuclear, Hospital de La Santa Creu I Sant Pau, Universitat Autònoma de Barcelona, Barcelona, Spain; 6 Nuclear Medicine Unit, IRCCS Ospedale Policlinico San Martino, Genoa, Italy; 7 Department of Diagnostic Radiology, Linköping University Hospital, Linköping, Sweden; 8 Department of Medical Physics, Linköping University Hospital, Linköping, Sweden; 9 National Institute of Nuclear Physics (INFN), Genoa section, Genoa, Italy; 10 Department of Nuclear Medicine, University Hospital, LMU Munich, Munich, Germany; 11 Department of Nuclear Medicine, Inselspital, University Hospital Bern, Bern, Switzerland; 12 Department of Neurosciences, Laboratory for Cognitive Neurology, Leuven, KU Belgium; 13 Neurology Department, University Hospitals Leuven, Leuven, Belgium; 14 Biomedical Research Institute, Hasselt University, Hasselt, Belgium; 15 Department of Neurology, University Medical Centre, Ljubljana, Slovenia; 16 Faculty of Medicine, University of Ljubljana, Ljubljana, Slovenia; 17 Department of Clinical Neurosciences, Geneva University Hospitals, Geneva, Switzerland; 18 LANVIE (Laboratoire de Neuroimagerie du Vieillissement), Department of Psychiatry, University Hospitals, Geneva, Switzerland; 19 Department of Neurology, Alzheimer Center, Amsterdam, The Netherlands; 20 Department of Radiology & Nuclear Medicine, Amsterdam UMC, location VUmc, Amsterdam, The Netherlands; 21 Neurology Unit, Department of Clinical and Experimental Sciences, University of Brescia, Brescia, Italy; 22 Parkinson’s Disease Rehabilitation Centre, FERB ONLUS – S. Isidoro Hospital, Trescore Balneario, BG Italy; 23 Department of Health Sciences, University of Genoa, Genoa, Italy; 24 Centre for Age-Related Medicine (SESAM), Stavanger University Hospital, Stavanger, Norway; 25 Department of Old Age Psychiatry, Institute of Psychiatry, Psychology and Neuroscience, King’s College London, London, UK; 26 Department of Neuroscience (DINOGMI), University of Genoa, Genoa, Italy; 27 Clinical Neurology, IRCCS Ospedale Policlinico San Martino, Genoa, Italy; 28 Division of Nuclear Medicine and Molecular Imaging, University Hospitals of Geneva and NIMTLab, Faculty of Medicine, University of Geneva, Geneva, Switzerland; 29 Center for Medical Image Science and Visualization (CMIV), Linköping University, Linköping, Sweden

**Keywords:** Artificial intelligence, Deep learning, FDG PET, Alzheimer’s disease, Mild cognitive impairment, Dementia with Lewy bodies

## Abstract

**Purpose:**

The purpose of this study is to develop and validate a 3D deep learning model that predicts the final clinical diagnosis of Alzheimer’s disease (AD), dementia with Lewy bodies (DLB), mild cognitive impairment due to Alzheimer’s disease (MCI-AD), and cognitively normal (CN) using fluorine 18 fluorodeoxyglucose PET (18F-FDG PET) and compare model’s performance to that of multiple expert nuclear medicine physicians’ readers.

**Materials and methods:**

Retrospective 18F-FDG PET scans for AD, MCI-AD, and CN were collected from Alzheimer’s disease neuroimaging initiative (556 patients from 2005 to 2020), and CN and DLB cases were from European DLB Consortium (201 patients from 2005 to 2018). The introduced 3D convolutional neural network was trained using 90% of the data and externally tested using 10% as well as comparison to human readers on the same independent test set. The model’s performance was analyzed with sensitivity, specificity, precision, F1 score, receiver operating characteristic (ROC). The regional metabolic changes driving classification were visualized using uniform manifold approximation and projection (UMAP) and network attention.

**Results:**

The proposed model achieved area under the ROC curve of 96.2% (95% confidence interval: 90.6–100) on predicting the final diagnosis of DLB in the independent test set, 96.4% (92.7–100) in AD, 71.4% (51.6–91.2) in MCI-AD, and 94.7% (90–99.5) in CN, which in ROC space outperformed human readers performance. The network attention depicted the posterior cingulate cortex is important for each neurodegenerative disease, and the UMAP visualization of the extracted features by the proposed model demonstrates the reality of development of the given disorders.

**Conclusion:**

Using only 18F-FDG PET of the brain, a 3D deep learning model could predict the final diagnosis of the most common neurodegenerative disorders which achieved a competitive performance compared to the human readers as well as their consensus.

## Introduction

Neurodegenerative dementias have a huge negative impact on the healthcare systems globally, especially with increasing older population. According to the World Health Organization, there are 50 million persons around the world suffering from dementia and 10 million new cases are anticipated every year [1]. Alzheimer’s disease, which is considered to be the most common neurodegenerative disorder, accounts for approximately 60% of all dementia [2]. Dementia with Lewy bodies (DLB) is another common neurodegenerative disorder, accounting for up to 30% of all cases of dementia [3] and is often misdiagnosed and unrecognized [4]. Mild cognitive impairment (MCI) is a prodromal form of dementia, defined by cognitive impairment not interfering with activities of daily living, leading to AD, DLB, or other degenerative dementias [5, 6]. The diagnosis of such disorders is challenging, even for experienced neurologists, making the decision of the use of the appropriate treatment difficult in some cases. Therefore, physicians use diagnostic tests such as neurofunctional imaging in order to provide more accurate clinical assessments [7]. 18F-FDG PET scans, which measure cerebral glucose metabolism, have been reported as a useful biomarker for the discrimination of the above-mentioned neurodegenerative disorders [8].

Deep learning (DL) methods have recently gained more popularity in medical image analysis and in specific in neurodegenerative diseases [9–11]. This wide recognition is due to its capability to learn complex representations in imaging data that are not easily detectable by humans [12], diminishing the need of manual feature extraction (compared to traditional machine learning techniques) and detecting the effective features automatically [13].

Most DL models applied in neurodegenerative diseases mainly focus on binary [13, 14] or classify multiple stages of AD from no dementia to moderate AD on 2D scans [9, 15]. However, the utility of such models is limited to the AD population solely, which makes them unable to discriminate from non-AD patterns. In addition, it is difficult to validate their robustness in the presence of non-AD dementias. The proper diagnosis of dementia patients requires going beyond binary classification and at least recognizing the differences among cognitively normal (CN), MCI and other types of dementia, especially the most common ones such as AD and DLB considering the 3D nature of such scans.

This study introduces a 3D-CNN model that can predict the final clinical diagnosis of CN, MCI due to AD and patterns of some types of dementia which can represent a challenge in their differentiation for the average reader, like AD and DLB. We hypothesized that a well-designed 3D-CNN model could take the advantage of the 3D 18F-FDG PET scans, detect features or patterns in these kinds of patients, and match or even provide better results than the experienced human readers, improving the final diagnostic classification of individuals. The model interpretation results indicate specific brain regions which makes the most discriminations among the included neurodegenerative disorders that confirm the findings from the clinical studies.

## Material and method

### Data acquisition

**Fig. 1:**
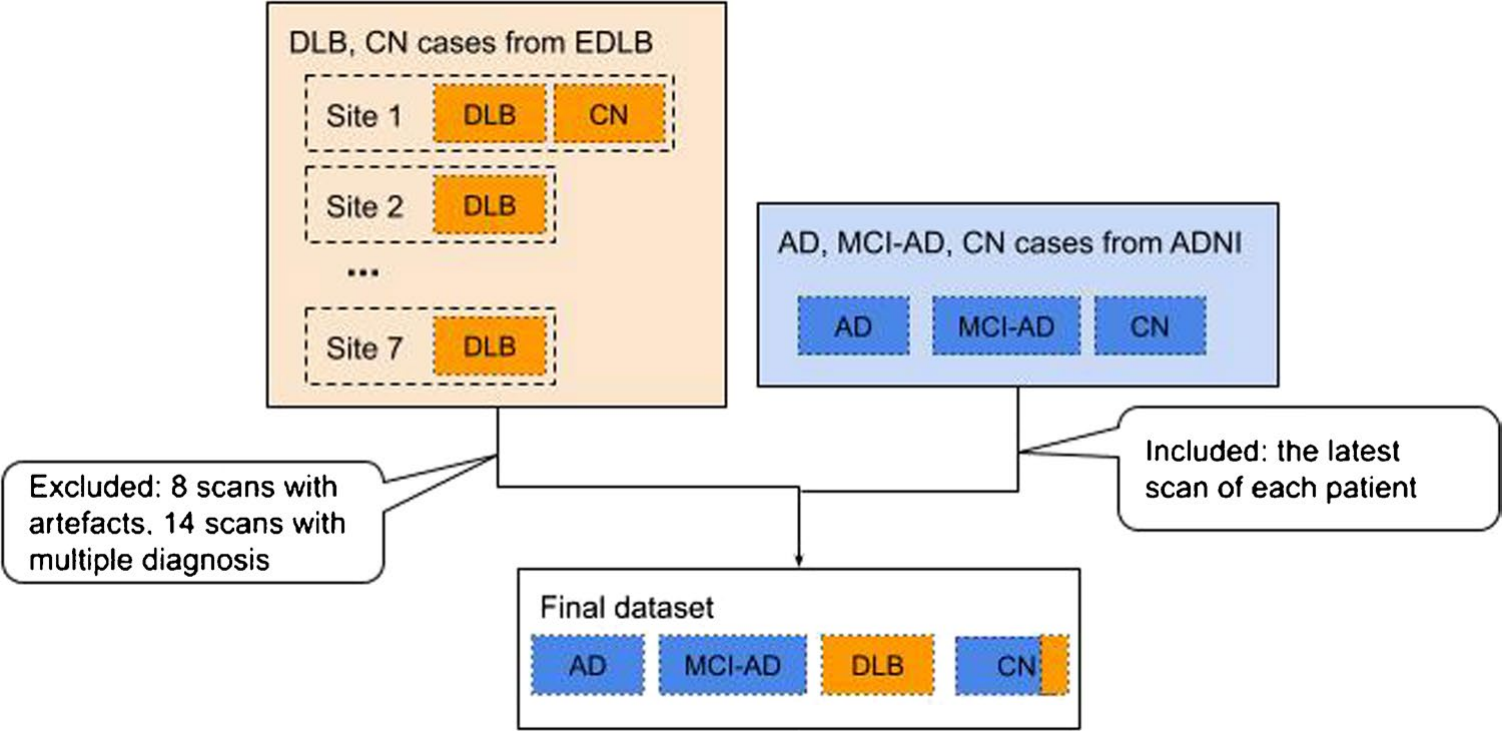
Inclusion and exclusion criteria for the datasets used. Since Alzheimer’s disease neuroimaging initiative (ADNI) includes a larger set of Alzheimer’s disease (AD), mild cognitive impairment due to AD (MCI-AD), and cognitively normal (CN), we included those that have no artefacts up to 200 cases per each disorder (except dementia with Lewy bodies (DLB) which the European DLB consortium (EDLB) provided)

The retrospective scans were collected from two different sources (Fig. 1). The anonymized scans from patients with probable DLB were collected from the European DLB (EDLB) Consortium,^1^ which has its core laboratory at Genoa, Italy having the local institutional ethics committee approvals including the transfer of fully anonymized imaging brain 18 F-FDG PET scans. The scans were performed according to the European Association of Nuclear Medicine (EANM) guidelines [16] from February 2005 to September 2018. Recruited patients were referred to and assessed at outpatient clinics including memory, movement disorders, geriatric medicine, psychiatric, and neurology clinics as previously described in [17]. Given the retrospective nature of the present study, diagnosis of probable DLB was originally made according to diagnostic criteria for probable DLB as defined by [18].

The EDLB also provided several normal cases that we added to the CN database. In order to have comparable sample sizes with DLB, up to 200 scans with AD, MCI, and CN were downloaded from the Alzheimer's disease neuroimaging initiative (ADNI)^2^ [19] across ADNI-1, ADNI-2, ADNI-3, and ADNI-GO (Grand Opportunities) studies from December 2005 to March 2020. The ADNI was launched in 2003 as a public–private partnership, led by Principal Investigator Michael W. Weiner, MD. The primary goal of ADNI has been to test whether serial magnetic resonance imaging (MRI), positron emission tomography (PET), other biological markers, and clinical and neuropsychological assessment can be combined to measure the progression of MCI and early AD. Detailed 18 F-FDG PET imaging protocols can be found at ADNI*.*^3^ Data regarding the patient’s final diagnosis were downloaded from the ADNI web portal. For each case, the latest scans were included. ADNI also provides information regarding the conversions from MCI to AD. Therefore, the MCI cases are confined to MCI-AD where the latest scans of the MCI cases before conversion to AD during the follow-up period were included in this study.

For both datasets, final clinical diagnosis was used as the ground truth label. Ninety percent of the final dataset (684 cases) was used for model training and internal validation. The remaining 10% (73 cases) was used as an independent test set for the model and comparison of the reader’s clinical interpretations.

### Data preprocessing

The original DICOM/NIFTI formats were used. The PET scans were spatially normalized to match the International Consortium of Brain Mapping template [20] and then skull stripped using MATLAB R2016a^4^ and SPM12*.*^5^ The probability maps of gray matter, white matter, cerebrospinal fluid, bone, and soft tissue/air were extracted. The skull stripping was done by retaining the voxels with high probability of being gray matter, white matter, or cerebrospinal fluid while discarding those likely being bone and soft tissue/air. The normalized and skull stripped scans were then visually inspected to assess their normalization quality and ensure that the spatial normalization converged to an acceptable solution. All the brains were positioned approximately in the center of the volume.

The first 10 layers as well as the last 9 layers of each scan were excluded as they contain very small objects, resulting in having a 3D volume of (95 × 79 × 60). Since scans are from various sites, feature-wise normalization was performed using image data preprocessing library in Keras*,*^6^ i.e., intensities of range [0,1]. Particularly, we treated each scan as a sequence of 2D images along the axial plane. We applied feature-wise normalization for each scan separately such that each 3D voxel was normalized by subtracting feature-specific mean then dividing by the feature-specific standard deviation per each scan.

### Model training

**Fig. 2:**
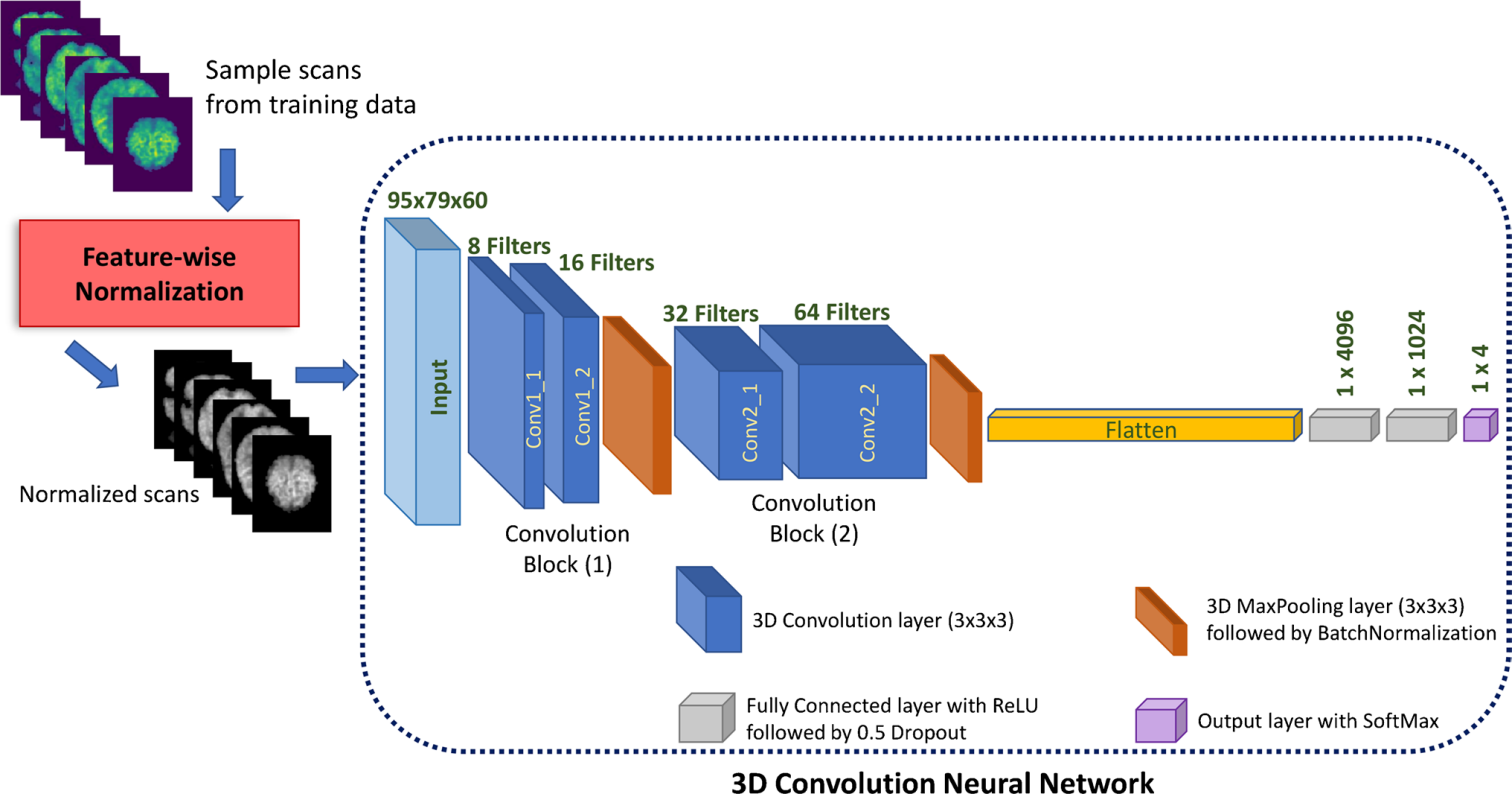
The 3D convolutional neural networks architecture of the introduced model. The model utilizes 3D 18F-FDG PET scans after being normalized via feature-wise normalization. We consider the input as a sequence of 2D images obtained along the axial plane from 18F-FDG PET scans

The 3D-CNN model is designed with reference to the architecture of VGG16 CNN [21] containing 2 convolutional blocks with 4 convolutional layers and a filter of size 3 × 3 × 3 across all convolution layers (Fig. 2). The model development and training were conducted using Keras library on a computer with Linux Ubuntu 18.09 operating system, one Nvidia Quadro GV100 GPU card with 32 GB of memory, and 36 CPU core Xenon with 128 GB of memory.

We performed end-to-end training using mini-batches of size 6 and Adadelta optimizer with 0.01 learning rate for 50 epochs. Dropout layers with 0.5 rate are used as a regularization method, forcing the network to learn more robust features. To prevent the model from overfitting, an early stopping condition was used by monitoring the validation loss in order to end the model training when the model performance stops improving (i.e. less than 0.0001 change in validation loss for 10 epochs).

The model training was performed through 20 rounds of *k*-fold cross validation with k ∈ [2, 10] on the training set and then accuracy is reported with confidence intervals (CI). The model with the highest validation accuracy is chosen for further fine-tuning using the training set with a stochastic gradient descent optimizer, 0.0001 learning rate, and 0.9 momentum for 50 epochs.

### Model interpretation and visualization

To visualize the attention of the network towards a specific class, we performed an occlusion experiment [22] for all four classes in the training dataset, where a volume of 6 × 5 × 5 is removed from the normalized scan with a stride of 2 for all 3 directions. The results show the cross-entropy response of the network given such occluded data as a function of the position of the occlusion box. The assumption is that when ignoring a relevant region for the correct classification, the cross-entropy response will be high. The maps are then projected using a mosaic of the slices 5 to 54 (to create a 7 × 7 grid) on the axial direction and over layered with the average brain. To visualize the metabolism patterns within each clinical diagnosis related to the highlighted brain regions in the occlusion heatmaps, the average normalized brain scan per each class is calculated over all available cases.

UMAP is a type of dimensionality reduction algorithm [23] to project the high-dimensional dataset into a 2-dimensional plane for easy visualization while preserving the relative closeness of data points. We used the unsupervised UMAP to visualize (1) the original normalized data and (2) the extracted features by 3D-CNN model (before the classification layer).

### Clinical interpretations

Four board-certified nuclear medicine physicians, R1, R2, R3, and R4, with 16, 13, 8, and 3 years of experience, respectively, performed independent interpretations of the independent test set (73 cases). The scans were available in axial, sagittal, and coronal views illustrated using Papaya.js volume viewer^7^ to the readers via a secure portal using their given credentials. Readers could log in whenever they want, interact with the viewer, and insert their readings including their diagnosis (among the four classes) into the portal. Only scans were visible to the readers (unlike natural clinical situations), the same as what had been used to train the deep learning model. The inter-rater agreement among the four readers using Fleiss’s kappa [24] is reported.

### Evaluations

For the external validation, receiver operating characteristic (ROC) curves of the model on the independent test set (i.e., 10% hold-out data) were plotted and the area under the ROC curve (AUC) was calculated with 95% CI.

For each scan in the independent test set, the majority voting of readers was taken as the consensus clinical diagnosis. In case of no consensus, the labels are scattered among the annotated labels, e.g., if an AD case is labeled as AD by two readers, MCI-AD by one reader, and CN by one reader, we calculate it as 0.5, 0.25, and 0.25 for AD, MCI-AD, and CN respectively. The sensitivity and specificity of readers’ performance and their consensus were plotted in the same ROC space. Sensitivity, specificity, precision, F1 score, and the confusion matrix with discussion on the misdiagnosed cases were reported for both the model and the consensus of human readers. Cohen’s kappa was calculated among consensus diagnosis and model predicted diagnosis.

### Model robustness

In order to investigate the model sensitivity and robustness to other similar dementia (i.e., something that the model has not been trained for), 18F-FDG PET scans for eight frontotemporal lobar degeneration (FTLD) cases were downloaded from the frontotemporal lobar degeneration neuroimaging initiative (FTLDNI) database. FTLDNI was funded through the National Institute of Aging, and started in 2010. The primary goals of FTLDNI were to identify neuroimaging modalities and methods of analysis for tracking frontotemporal lobar degeneration (FTLD) and to assess the value of imaging versus other biomarkers in diagnostic roles.^8^

The FTLD scans were pre-processed with the same procedure mentioned before and using the proposed 3D-CNN model; we plot the UMAP as well as the occlusion maps besides the output of the model for these eight FTLD cases.

### MMSE-based classification

To perform further classification analysis and enhance the translational potential of the proposed model, a new model with different split strategies for training and testing datasets was developed using MMSE scores. MMSE score is used to assess changes to patients suffering from dementia such low score indicates severe dementia while high score indicates early or mild conditions of dementia. Thus, scans associated with high MMSE scores can be challenging for diagnosis. We performed data stratification according to MMSE to force the model to get trained on severe cases and tested on mild ones. After sorting the cases in each clinical diagnostic class, 80% (439 cases with low MMSE score) were used for training and remaining 20% (112 cases having high MMSE scores in each category) for testing. We trained the model using KFCV for 10 rounds. Several performance metrics including accuracy, ROC curve, AUC, classification results, and UMAP visualization of dataset using the new model are reported.

## Results

### Demographics

Table 1 summarizes the demographics and mini-mental state examination (MMSE) scores of the two datasets used in this study: EDLB and ADNI, as well as the train and test set distributions. The dataset consisted of 757 cases including 200 AD (from ADNI), 200 MCI-AD (from ADNI), 157 DLB (from EDLB), and 200 CN (156 cases from ADNI and 44 cases from EDLB).

**Table 1:** Demographics of datasets. (a) Alzheimer’s disease neuroimaging initiative (ADNI) and the European DLB consortium (EDLB) datasets, (b) train set (used for model training and internal validation), and the independent test set (used for model testing and comparison to readers)

a: Datasets					
ADNI set		No. (percentage)	Average age (year)		
Clinical diagnosis	No. cases	Female sex	Male	Female	MMSE score
AD	200	72 (36.0%)	76.7 ± 8.2 (56–92)	74.0 ± 7.8 (56–89)	22.3 ± 3.3 (8–30)
MCI-AD	200	62 (31.0%)	78.6 ± 7.0 (59–91)	76.6 ± 6.9 (57–96)	26.0 ± 3.9 (10–30)
CN	156	62 (39.7%)	77.5 ± 5.4 (62–89)	78.3 ± 5.0 (64–87)	29.1 ± 1.4 (18–30)
All-ADNI	556	196 (35.2%)	77.6 ± 7.1 (56–92)	76.2 ± 7.0 (56–96)	
EDLB set					
DLB	157*	59 (37.5%)	73.3 ± 7.2 (53–91)	74.8 ± 6.4 (58–86)	22.4 ± 4.7 (5–30)
CN	44**	22 (50.0%)	70.1 ± 10.3 (48–84)	67.5 ± 9.2 (50–83)	
All-EDLB	201	81 (40.2%)	72.7 ± 7.9 (48–91)	72.9 ± 7.9 (50–86)	
Total	757	277 (36.6%)	76.4 ± 7.6 (48–92)	75.2 ± 7.4 (50–96)	
b: Train and test sets					
Train set:		No. (percentage)	Average age (year)		Source (percentage)
Clinical diagnosis	No. cases (percentage)	Female sex	Male (range)	Female (range)	EDLB
AD	178 (89%)	67 (37.6%)	76.9 ± 8.3 (56–91)	74.1 ± 7.7 (59–89)	0 (0.0%)
MCI-AD	194 (97.0%)	60 (30.1%)	78.6 ± 6.8 (60–91)	76.6 ± 7.0 (57–96)	0 (0.0%)
DLB	136 (86.4%)	52 (38.2%)	73.7 ± 7.0 (55–91)	74.8 ± 6.2 (58–86)	136 (100%)
CN	176 (88%)	71 (40.3%)	76.5 ± 6.6 (48–89)	75.4 ± 8.2 (50–87)	34 (19.3%)
All-train set	684 (90.4%)	250 (36.5%)	76.7 ± 7.4 (48–91)	75.2 ± 7.4 (50–96)	170 (25.2%)
Test set:					
AD	22 (11%)	5 (22.7%)	75.7 ± 8.4 (57–92)	71.6 ± 10.0 (56–82)	0 (0.0%)
MCI-AD	6 (3%)	2 (33.3%)	78.0 ± 14.6 (59–91)	75.5 ± 2.1 (74–77)	0 (0.0%)
DLB	21***(13.3%)	7 (33.3%)	70.5 ± 8.3 (53–87)	75.5 ± 8.5 (67—86)	21 (100%)
CN	24 (12%)	13 (54.1%)	72.0 ± 10.8 (55–85)	75.9 ± 6.1 (65–85)	10 (41.6%)
All-test set	73 (9.6%)	27 (37.0%)	73.5 ± 9.6 (53–92)	75.9 ± 7.2 (56–86)	31 (42.4%)

^*^15 cases with no registered MMSE score, where one of them reported language barrier as the reason

^**^No MMSE score collected for normal cases in EDLB

^***^One DLB case contained no age-sex information

*AD*, Alzheimer disease; *MCI-AD*, mild cognitive impairment due to AD; *DLB*, dementia with Lewy bodies; *CN*, cognitively normal

The average age of the patients was 77.6 years for men (between 56 to 92 years old) and 76.2 years for women (between 56 and 96 years old) in the ADNI dataset. In the EDLB set, the average age for men was 72.7 (between 48 to 91 years old) and 72.9 for women (between 50 and 86 years old). The overall percentage of women in the ADNI set was 35.2% (196 of 556), and in the EDLB set, was 40.2% (81 of 201).

Initially 200 scans (50 per each class) were sampled using stratified random sampling as the independent test set; but eventually 73 cases were read by all four readers.

### Clinical interpretations

Fleiss’s kappa among four readers was 0.19 when discriminating between the diagnoses of AD, MCI-AD, DLB, and CN solely based on metabolic patterns, which is considered as a *slight agreement* [25]. There were 10 cases in which there was no majority voting among readers (two AD, two CN, five DLB, and one MCI-AD cases). In 8 of these 10 cases, the correct clinical diagnosis was among the readers’ labels, meaning that two readers could diagnose the correct disorder while the other two voted for another disorder. The consensus accuracy of the readers was 0.57, and it is higher than each individual reader. The accuracies of R1, R2, R3, and R4 are 0.56, 0.50, 0.46, and 0.39, respectively which are positively associated with the readers’ experience.

More detailed readers’ labeling information is provided in Table 2. The Fleiss’ kappa is also calculated per each disorder to illustrate the inter-rater agreements in detail. Out of the 24 labels from the four readers for 6 MCI-AD cases, there were 9 (37.5%) CN, 9 MCI-AD, 4 (16.7%) AD, and 2 (8.3%) DLB, showing MCI-AD is commonly misdiagnosed as CN, which is very common in the clinical interpretation [26]. There was only one CN case where all the readers voted for CN, and in the remaining ones, votes were scattered mainly among MCI-AD and CN, that explains very low agreement in CN cases.

**Table 2:** Confusion matrix: labels of readers and the model for the independent test set (the highest label ranked by readers per each disorder (in each row) is shown in bold. The diagonal numbers are the true positives shown in underlined

Readers’ labels						
Actual label	AD*	MCI-AD*	DLB*	CN*	Sum (no. cases)	Fleiss’s kappa
AD	**36 (40.9%)**	27 (30.6%)	6 (6.8%)	19 (21.6%)	88 (22)	0.21
MCI-AD	4 (16.7%)	**9 (37.5%)**	2 (8.3%)	**9 (37.5%)**	24 (6)	0.07
DLB	19 (22.6%)	11 (13.1%)	**41 (48.8%)**	13 (15.5%)	84 (21)	0.04
CN	4 (4.2%)	27 (28.1%)	10 (10.4%)	**55 (57.3%)**	96 (24)	-0.02
Sum	63 (21.6%)	74 (25.3%)	59 (20.2%)	96 (32.9%)	292 (73)	0.19
Model Labels						
Actual label	AD*	MCI-AD*	DLB*	CN*	No. cases	Cohen’s kappa model vs. consensus
AD	**20 (91%)**	1 (4.5%)	0	1 (4.5%)	22	0.13
MCI-AD	**3 (50%)**	1 (16.7%)	0	2 (33.3%)	6	0.21
DLB	0 (%)	1 (4.8%)	**18 (85.7%)**	2 (9.5%)	21	0.27
CN	1 (4.2%)	2 (8.3%)	0	**21 (87.5%)**	24	0.68
Sum	24 (32.9%)	5 (6.8%)	18 (24.7%)	26 (35.6%)	73	0.54

^*^Numbers in parentheses show the percentage of the whole labels collected for all scans per each disorder

*AD*, Alzheimer disease; *MCI-AD*, mild cognitive impairment due to AD; *DLB*, dementia with Lewy bodies; *CN*, cognitively normal

Readers had the highest agreement in AD, with 0.21 as the Fleiss’s kappa. In three of 22 AD cases, all four readers correctly labeled them as AD and in other three cases, three readers converged to AD. Lastly, there was one DLB case where there was no agreement among the readers.

 Performance metrics for readers are shown in Table 3. In general, readers have higher performance metrics in DLB compared to other clinical diagnoses. Readers are performing very high in ruling out cases having no DLB (100%); all of 52 non-DLB (i.e., MCI-AD, AD, or CN) labels were truly non-DLB. On the contrary, their performance metric for MCI-AD is relatively low, which is in line with the results from [10].

**Table 3:** Performance metrics for the proposed deep learning model and the consensus of the readers (bold values are the highest between readers consensus vs. model performance, underlined values illustrate similar performance)

Metric	Sensitivity *	Specificity *	Precision *	F1 score	No. cases
Consensus of the readers					
AD	0.47 (10.5/22)	0.90 (46.25/51)	0.68 (10.5/15.25)	0.56	22
MCI-AD	**0.25** (1.5/6)	0.75 (50.25/67)	0.08 (1.5/18.25)	0.12	6
DLB	0.63 (13.25/21)	**1.0** (52/52)	**1.0** (13.25/13.25)	0.77	21
CN	0.70 (17/24)	0.81 (39.75/49)	0.64 (17/26.25)	0.67	24
Model					
AD	**0.91** (20/22)	**0.92** (47/51)	**0.83** (20/24)	**0.87**	22
MCI-AD	0.17 (1/6)	**0.94** (63/67)	**0.20** (1/5)	**0.18**	6
DLB	**0.86** (18/21)	**1.0** (52/52)	**1.0** (18/18)	**0.92**	21
CN	**0.88** (21/24)	**0.90** (44/49)	**0.81** (21/26)	**0.84**	24

^*^Numbers in parentheses are the number of cases (raw data) used to calculate the metric

In case of no consensus, the labels are scattered among the annotated labels, e.g. if an AD case is labeled as AD by two readers, MCI-AD by one reader, and CN by one reader, we calculate it as 0.5, 0.25, and 0.25 for AD, MCI-AD, and CN respectively

*AD*, Alzheimer disease; *MCI-AD*, mild cognitive impairment due to AD; *DLB*, dementia with Lewy bodies; *CN*, cognitively normal

### Model training

**Fig. 3:**
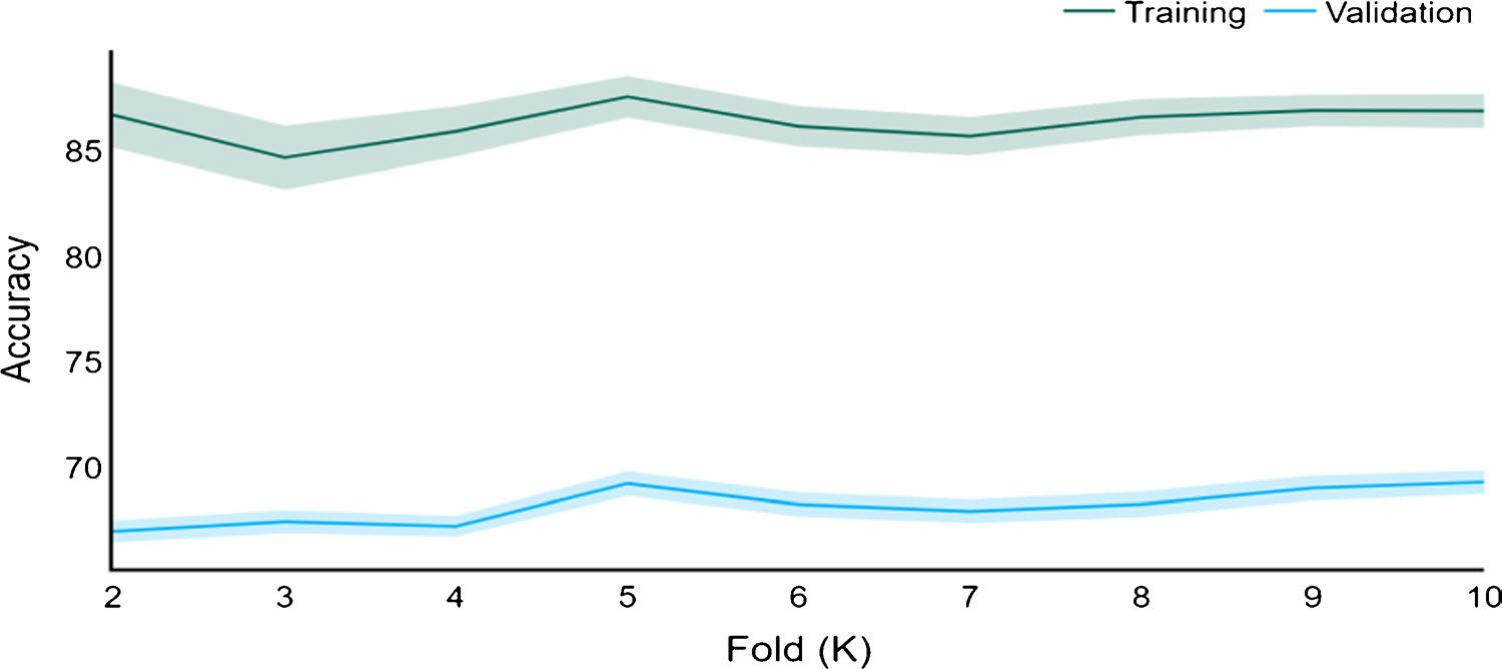
Confidence intervals (95% CI) for training and validation accuracy during *k*-fold cross validation. KFCV, *k*-fold cross validation

Figure 3 depicts the 95% confidence intervals for training and validation accuracy for each *k*. The highest validation accuracy of 78.9% was achieved with 608 samples and the validation accuracy was computed using the remaining 76 samples of the training set.

### Evaluations

**Fig. 4:**
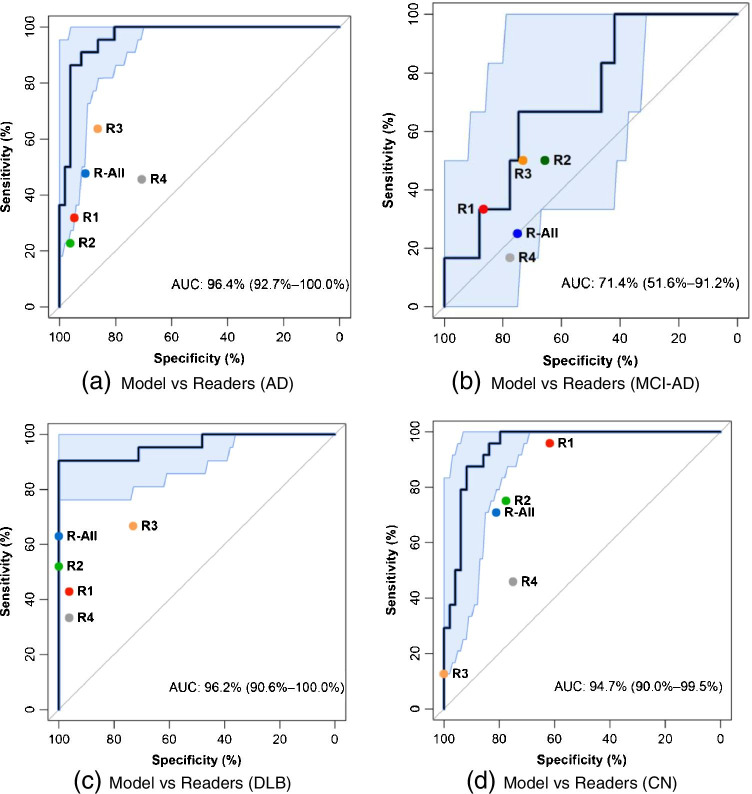
Receiver operating characteristic (ROC) curves with 95% confidence interval (CI) on the test set for **a** Alzheimer disease (AD), **b** mild cognitive impairment due to AD (MCI-AD), **c** dementia with Lewy bodies (DLB), and **d** cognitively normal (CN). (R-All indicates the consensus labeling among the four readers, R1-4, reader 1–4)

The performance metrics for the trained model is shown in Table 3, as well as the readers’ performance metrics. The ROC curves of the model and the readers are shown in Fig. 4. The AUC for prediction of DLB, AD, MCI-AD, and CN was 96.2%, 96.4%, 71.4%, and 94.7% respectively. Both the model and readers are performing higher in DLB cases and lower in MCI cases. The model reached a perfect performance in DLB cases with 86% sensitivity (18 out of 21 DLB cases were detected), 100% specificity (52 non-DLB cases by the model were correctly ruled out), and 100% precision (18 cases labeled as DLB were correctly classified), and F1 score 92%. The proposed model performed better than all the readers and their consensus, in some cases even statistically significant. As depicted in Fig. 4, some readers have higher sensitivity in some disorders while others have higher specificity. For instance, R1 has higher sensitivity in diagnosing CN, while R3 has higher specificity in the same disorder.

Cohen’s kappa among consensus diagnosis and model predicted label was 0.54, which is considered as moderate agreement. Among 13 misdiagnosed cases by the model, there were 5 MCI-AD cases, 2 AD, 3 CN, and 3 DLB cases as illustrated in Table 2. We looked into these cases and compared them to the readers’ labeling. In MCI-AD cases, one case was correctly diagnosed by both the model and the readers, and the remaining 5 cases, all were misdiagnosed both by the consensus of the readers and the model, that explains the high agreement between consensus of the readers and the model (Cohen’s kappa 0.68). In total, among these 13 model-misdiagnosed cases, 11 cases were also consensus-misdiagnosed, and 6 cases were similarly consensus-misdiagnosed to the same disorder.

### Model interpretation and visualization

**Fig. 5:**
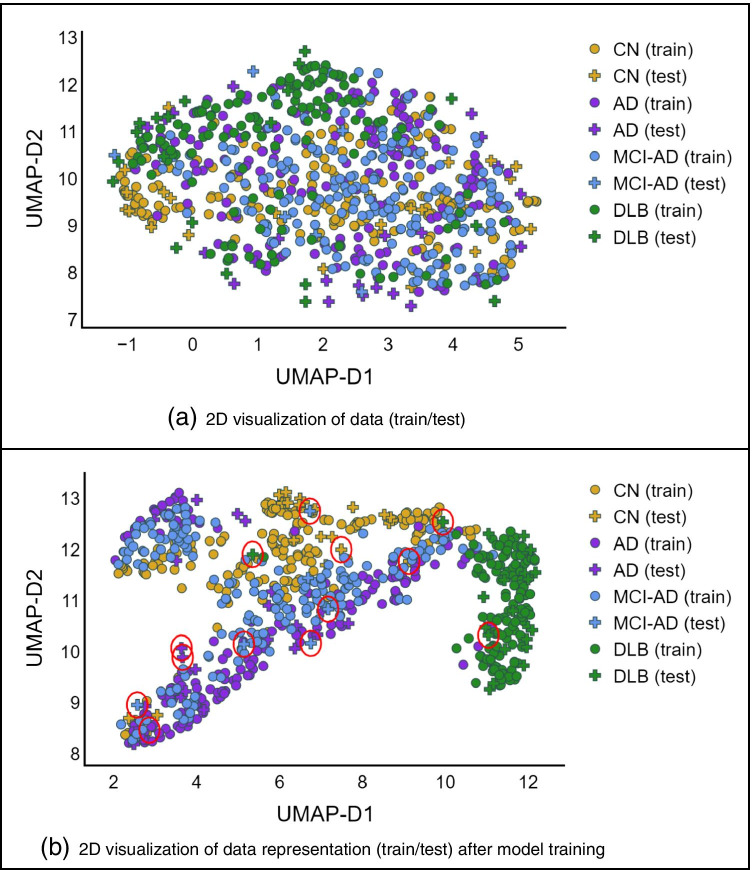
Uniform manifold approximation and projection (UMAP) visualization of the data: **a** shows the visualization of the original input data and **b** shows the map of the features extracted from the proposed model, the layer before the classifier layer. The red circles denote the misclassified testing samples. (AD, Alzheimer disease; MCI-AD, mild cognitive impairment due to AD; DLB, dementia with Lewy bodies; CN, cognitively normal; UMAP-D, uniform manifold approximation and projection dimension)

As shown in UMAP visualizations in Fig. 5b, DLB cases were separated compared to Fig. 5a and it is explaining the good performance of the model due to the relevant extracted features. The other interesting pattern in this figure is the distribution of cases from CN to MCI-AD and then to AD, which is happening also in reality. DLB cases are very well separated and cases from CN to AD are spread from CN to MCI-AD and to AD, which explains the development of AD. The extracted features of the proposed model were able to separate these four classes well enough, although using unsupervised UMAP.

The test cases with red circles in Fig. 5b are the misclassified cases by the model. They are mainly those cases that have happened to be in the middle of the wrong class or in the borders of two classes. It is worth mentioning again that the ground truth labels for the whole dataset (both the ADNI and EDLB) are the final clinical diagnosis obtained from these sources and we are not aware if necropsy evaluation has been performed in any of those cases.

**Fig. 6:**
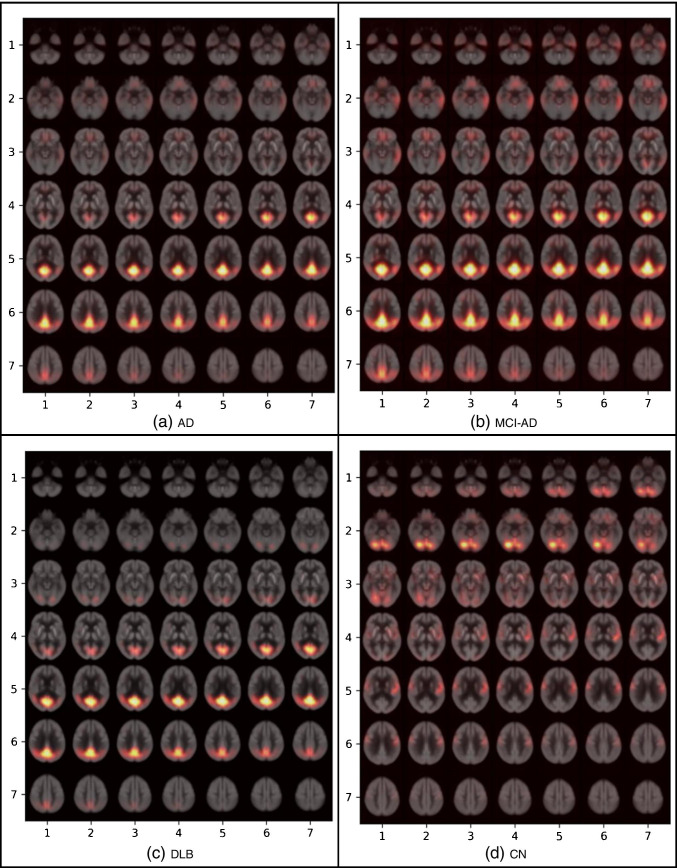
Results of the occlusion experiments for **a** Alzheimer disease (AD), **b** mild cognitive impairment due to AD (MCI-AD), **c** dementia with Lewy bodies (DLB), and **d** cognitively normal (CN). The results are projected by creating a mosaic of slices in the axial direction. The cross-entropy maps have been over layered with the average brain

The results of the occlusion experiment which indicated the network attention are illustrated in Fig. 6. The highlighted regions in each disorder indicate which brain regions were of more attention in the proposed model in its prediction. In AD (Fig. 6a), the posterior cingulate cortex is the most discriminating region, slightly along with the temporal lobes and the anterior cingulate cortex. In MCI-AD (Fig. 6b), the most discriminating regions are similar to AD with more emphasis on the posterior cingulate cortex, the middle temporal gyrus, gyrus rectus/orbital gyri, and also on the parieto-occipital cortex. Furthermore, the posterior cingulate cortex is also taking an important role in differentiating DLB cases (Fig. 6c) besides the occipital cortex. And finally, in CN (Fig. 6d), the occipital cortex, the cerebellum, and slightly postcentral gyrus and striatum are the highlighted regions.

The posterior cingulate cortex is important for all the given neurodegenerative disorders, i.e., AD, MCI-AD, and DLB, and not in CN. It shows the pattern in this brain region makes the most difference in a cognitively normal brain compared to dementia-involved ones.

**Fig. 7:**
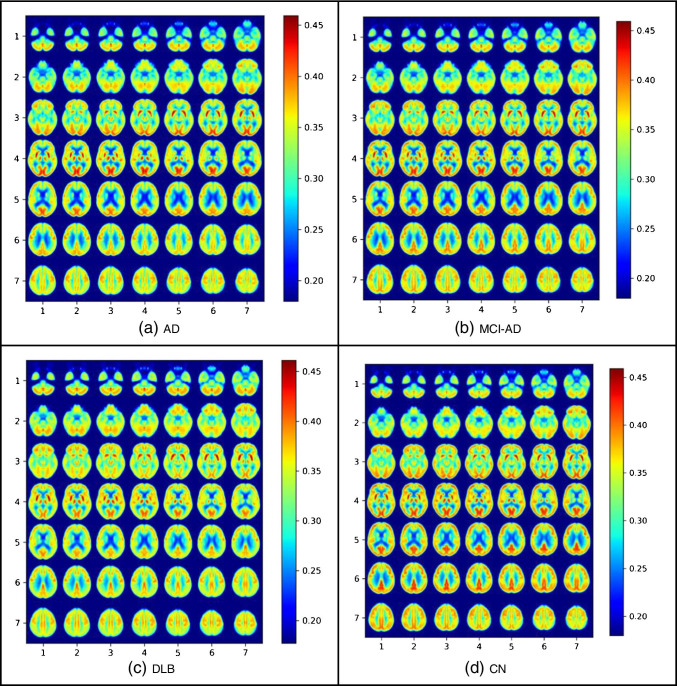
The average brain scan for all normalized cases with **a** Alzheimer disease (AD), **b** mild cognitive impairment due to AD (MCI-AD), **c** dementia with Lewy bodies (DLB), and **d** cognitively normal (CN)

The average over all normalized brain scans for each clinical diagnosis is illustrated in Fig. 7. AD (Fig. 7a) and MCI-AD (Fig. 7b) share similar metabolism patterns with MCI-AD in the highlighted regions as shown in Fig. 6b. The hypometabolism pattern in the posterior cingulate cortex differs the most among the different disorders as expected from Fig. 6.

### Model robustness

**Fig. 8:**
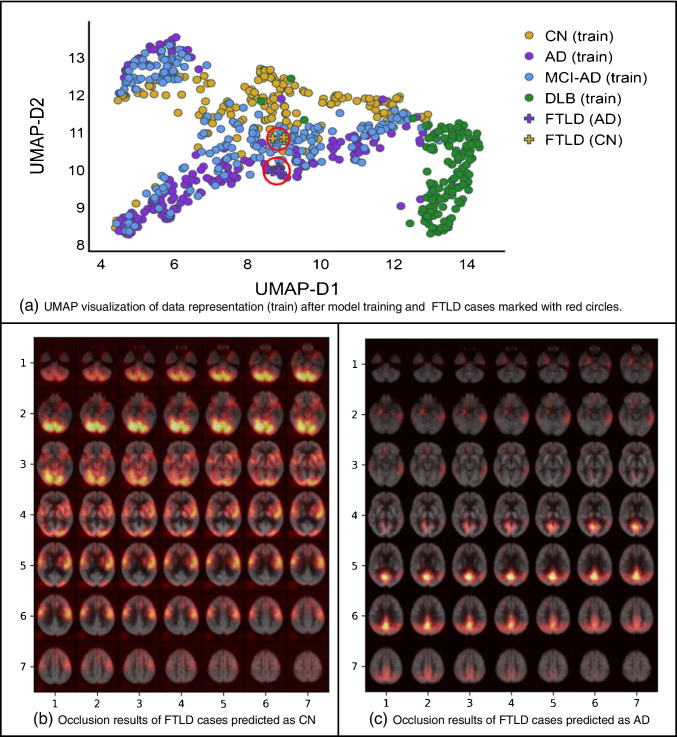
Results of FTLD cases, **a** the representation space with training dataset and adding new 8 cases with FTLD, **b** occlusion results of FTLD cases labeled as CN, and **c** occlusion results for FTLD cases labeled as AD

Among the eight FTLD cases, three cases were classified as CN while the remaining five cases were classified as AD by the model. Figure 8a shows the UMAP representation of the training set (similar to Fig. 5b with test cases excluded) where FTLD cases are also plotted. All the eight cases are mapped close to each other in the UMAP space. Interestingly, the generated representation reflects the similarity of FTLD cases with CN and AD cases and not to DLB. What is expected to observe in FTLD is low FDG uptake in the frontal and temporal lobes [29]. A patient with a chronic AD can eventually have involvement of the frontal lobes and look like a FTLD. These five FTLD cases are very probable to have involvement not only of the frontal and temporal lobes but even the parietal lobes might be involved. We performed the occlusion experiments using FTLD cases to investigate further the highlighted regions in these brain scans. As shown (see Fig. 8.b and 8.c), though there is a huge overlap in highlighted regions with previous results of AD/CN, FTLD cases show different intensities. Hence, the learned patterns by the model correspond to the metabolism patterns within each disorder.

### MMSE-based classification

**Fig. 9:**
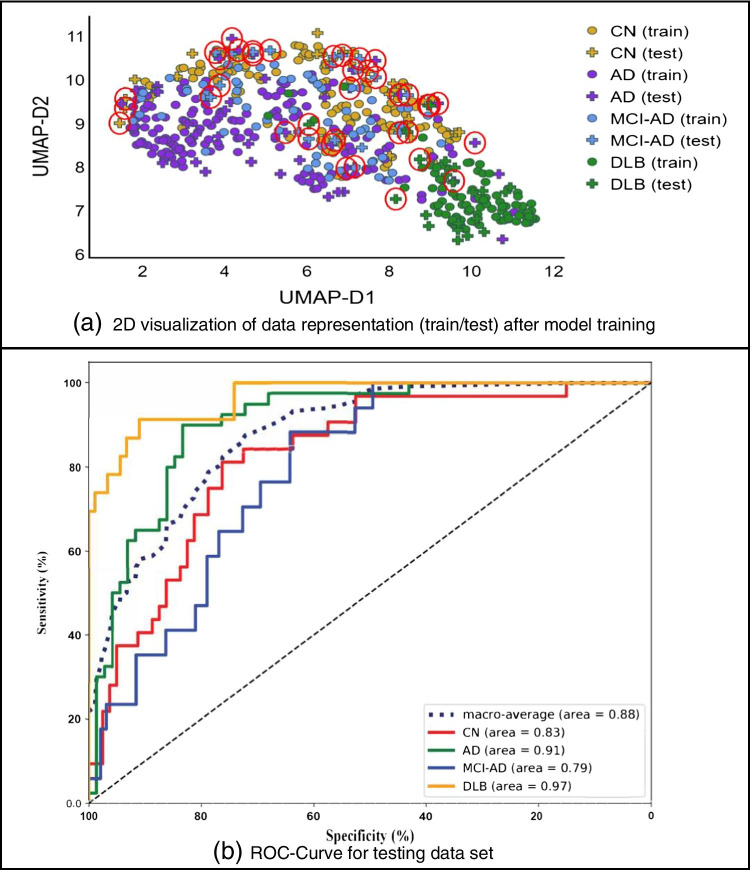
Results of training a new model with MMSE-based data split, **a** the UMAP visualization of training/testing datasets with misclassified test cases identified with red circles, **b** receiver operating characteristic (ROC) curve for model predictions with testing dataset

The new model trained on low MMSE scores achieved best accuracy 80%, 82%, 66% for training, validation, and testing accuracy, respectively. The validation accuracy is higher than the validation accuracy of the proposed model trained without MMSE stratification, while the testing accuracy is lower. The decay in performance is expected, due to the stratification that forces the model to get trained on easy cases and tested on hard cases to predict. The test set contained 112 cases, out of which 33 were misclassified. Figure 9a depicts the UMAP visualization of the new trained model, which conveys the same pattern as the UMAP visualization of the random split model shown in Fig. 5b with less clear borders that justifies the lower performance. The ROC curves and AUCs are shown in Fig. 9b. Compared to the random split results, no change in DLB and MCI-AD is observed, but AD and CN experienced a drop of 5 and 10% in AUC respectively.

**Fig. 10:**
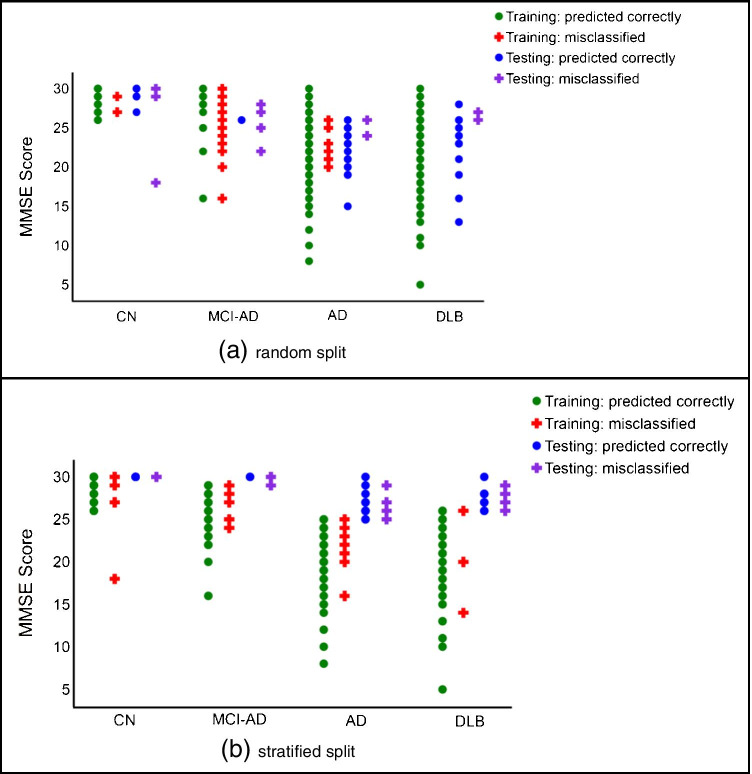
MMSE-based classification results: **a** random split, **b** stratified split where low MMSE scores are used for training and high ones for test

Figure 10 illustrates the MMSE scores of the classification results both in the random split (Fig. 10a) which is performed in design of the proposed model, and the stratified split where the same model is trained on low MMSE scores, and tested on high MMSE scores (Fig. 10b). In the random split, the misclassification errors happened both in low and high MMSE scores in CN and MCI-AD groups, while in AD and DLB, the few misclassified test cases occurred close to the high MMSE scores. However, in the stratified split, the misclassified test cases are not different from the correctly classified test cases in terms of MMSE score.

## Discussion

Today, nuclear medicine specialist physicians make pattern recognition decisions on FDG PET scans using visual and qualitative readings, which is complex and challenging and needs years of experience. In this study, we proposed a 3D-CNN model to predict the diagnosis based on 18F-FDG PET scans. The datasets were achieved from ADNI and EDLB. The performance of the model was shown to be robust across the studied disorders: DLB, AD, MCI-AD, and CN and also in comparison to the nuclear medicine readers. The proposed model reached a competitive performance compared to an experienced reader and also the consensus of them. With further validation with more diverse datasets and extending to include more similar disorders, the proposed model can be used as an augmentation to the provided 18F-FDG PET software, improving the diagnosis of such neurodegenerative disorders, especially in situations where there is missing presence of experienced physicians.

This study has several strengths and contributions that are briefly explained here. First, the dataset is relatively diverse since it contains cases from ADNI and EDLB sources, in specific the CN cases were a combination of both sources and DLB cases were from 7 different sites in Europe. The MCI cases were confined to those that further developed AD and not including MCI as a generic cognitive impairment. Second, the test set was relatively big (*n* = 73) compared to the similar studies, for example (*n* = 40) within the independent test set as reported in [10] and (*n* < 35) with tenfold cross validation as reported in [27]. The CN cases in the test set were selected balanced from the two sources ADNI and EDLB not to be biased towards ADNI which is more dominant in providing our CN cases.

Third, the dataset includes DLB, a non-AD disorder, as well as the AD family (AD and MCI-AD), and CN to make it more robust in the predicted diagnosis compared to similar studies which tried to discriminate different stages of AD, make the utility of their algorithm limited to AD patient population only.

And finally, the extracted patterns from the interpretation of the model show that the posterior cingulate cortex is playing an important role in discriminating these neurodegenerative disorders, i.e., AD, MCI-AD, and DLB, and not in CN. It shows the pattern in this brain region makes the most difference in a cognitively normal brain compared to dementia-involved ones. It also depicted AD and MCI-AD sharing the same affected brain regions. While in [10], model visualization with saliency map did not reveal a human interpretable imaging biomarker that appears influential for AD prediction.

Recently, substantial work in the area of applying DL methods has been done for the classification of different brain disorders [9–11]. However, most of the work has been performed using structural imaging of the brain and very little work has been presented by applying DL, particularly CNNs, using functional imaging, specifically 18F-FDG PET scans. In Table 4, we summarized the results obtained by recent DL studies for the diagnosis of AD and MCI using FDG PET [10, 14, 27, 28].

**Table 4:** Summary of the state-of-the-art studies applying deep learning (DL) using 18F-FDG PET for diagnosis of Alzheimer’s disease (AD) and mild cognitive impairment (MCI). We report the performance of DL models using classification accuracy (ACC), sensitivity (SEN), specificity (SPE), and area under the curve (AUC)

Reference	Summary	Disorders	Dataset specifications	ACC		SEN		SPE	AUC	
Ding et al. 2019 [10]	This work presented the success of adopting transfer learning for medical imaging by using InceptionV3 model pre-trained on ImageNet. The model was fine-tuned using 18F-FDG PET scans from ADNI (1921 imaging studies). However, the introduced method transformed 3D 18F-FDG PET scans into 2D grids which prevented generating human interpretable imaging to reveal model decisions for AD prediction	AD, MCI, non-AD/MCI	**ADNI** 2019 scans including: 484 AD 861 MCI 764 non-AD/MCI **Authors’ Institution** 40 imaging studies including: 7 AD 7 MCI 26 non-AD/MCI	(ADNI) AD						
				-	81%		94%			92%
				(ADNI) MCI						
				-	54%		68%			63%
				(ADNI) Non-AD/MCI						
				-	59%		75%			73%
				(AI) AD						
				-	100%		82%			98%
				(AI) MCI						
				–	43%		58%			52%
				(AI) Non-AD/MCI						
				–	35%		93%			84%
Huang et al. 2019 [14]	A 3D VGG network was introduced to predict the development of AD by processing multi-modality information via usage of both T1-MR and 18F-FDG PET images. The results showed a slight improvement in performance of classifying AD, MCI, and CN patients. For sake of comparison with other methods, we reported the results of the models that were trained using a single imaging source as well as multi-modality information	AD, MCI, AD	**ADNI** 2145 18F-FDG PET scans including: 647 AD 731 CN 767 MCI	(18 F-FDG PET) CN vs. AD						
				89.1%	90.2%		87.8%			92.7%
				(T1-MR) CN vs. AD						
				81.2%	79.3%		83.5%			83.7%
				(Multi-modality) CN vs. AD						
				90.1%	90.9%		89.2%			90.8%
				(Multi-modality) CN vs. pMCI						
				87.5%	90.8%		80.6%			87.6%
Liu et al. 2018 [27]	The model provided the classification using 2D CNN and recurrent neural networks (RNNs). The model treated 3D 18 F-FDG PET as a sequence of 2D images. Adopted DL methods captured the intra-slice and inter-slice features for the classification task. However, constructing 2D sequences representing different views increases the complexity of used DL models, thus limits the applicability of such framework on large datasets	AD, MCI, CN	**ADNI** 339 subjects including: 93 AD 146 MCI 100 CN	AD vs. CN						
				91.2%		91.4%		91%	95.3%	
				MCI vs. CN						
				78.9%		78.1%		80%	83.9%	
Shen et al. 2019 [28]	A deep belief network was used to extract feature representation of some extracted regions of the brain by employing region-growing algorithm. Additionally, a support vector machine classifier was used to identify patients in MCI who would progress on to AD (MCI-AD) and discriminate these from patients with stable MCI conditions (i.e., sMCI)	MCI-AD, sMCI	**ADNI** 109 18 F-FDG PET scans for MCI cases including: 47 MCI-AD 62 sMCI	MCI-AD vs. sMCI						
				86.6%	89.5%		82.5%			90.9%

*DL*, deep learning; *CN*, cognitively normal; *MCI-AD*, mild cognitive impairment due to AD; *sMCI*, stable MCI; *ADNI*, Alzheimer’s disease neuroimaging initiative

In summary, the proposed model takes the advantage of the 3D 18F-FDG PET scans and provides high predictive performance as well as strong generalizability with the diagnosis of multiple neurodegenerative disorders. Differently from existing methods, the presented model can distinguish cases of AD, CN, MCI-AD, and DLB with AUC of 96.4%, 94.7%, 71.4%, and 96.2%, respectively. The model robustness test over a few FTLD cases (which was not part of the training process), revealed that the learned metabolism by the model are relevant and consistent to the expected patterns.

One of the limitations of the study was that all AD and MCI-AD disorders were obtained from ADNI, which makes the robustness of the proposed model in these two cases limited to the clinical distribution of ADNI datasets. Furthermore, the number of MCI-AD cases in the independent test set was small. Performance dropped somewhat for the classification of MCI-AD cases, but this was analogous to the performance of the readers. Also, FDG PET may be normal in the MCI-AD stage where the diagnosis heavily relies on fluid biomarkers.

The second limitation is that the proposed model predicted the diagnosis based on 18F-FDG PET scans only, the same with the human readers in this study. But, in real practice, clinicians make the final decision based on several other clinical evaluations. We believe if other clinical evaluations of the patients are added to the model, the performance will even reach higher values. On the other hand, the proposed model is able to be embedded to the 18F-FDG PET software devices that nuclear medicine specialists are normally using without any extra patient information needed and still be able to discriminate among several neurodegenerative disorders with high performance.

Third, we only include DLB as a non-AD disorder. It is worth trying to include more neurodegenerative disorders to check the robustness of the algorithm in the presence of other similar diseases.

One of the future works alongside with providing solutions to the above-mentioned limitations will be to investigate integrating the proposed algorithm into clinical workflow as a decision support tool. We will look into how to add more explanations to the outcome of the provided model to increase transparency and trust.

## Data Availability

Part of data collection and sharing for this project was funded by the Alzheimer's Disease Neuroimaging Initiative (ADNI) (National Institutes of Health Grant U01 AG024904) and DOD ADNI (Department of Defense award number W81XWH-12–2-0012).

## Footnotes

1 https://www.ge.infn.it/wordpress/?page_id=77&lang=en.

2 adni.loni.ucla.edu.

3 http://adni.loni.usc.edu/methods/documents/*.*

4 https://www.mathworks.com/help/matlab/release-notes-R2016a.html.

5 https://www.fil.ion.ucl.ac.uk/spm/software/spm12/.

6 https://keras.io/api/preprocessing/image/*.*

7 https://github.com/rii-mango/Papaya.

8 For up-to-date information on participation and protocol, please visit http://memory.ucsf.edu/research/studies/nifd.

## References

[1] Dementia Key Facts [Internet]. WHO. 2020 [cited 2021 Apr 6]. Available from: https://www.who.int/news-room/fact-sheets/detail/dementia. Accessed Jan-March 2021.

[2] Ferri CP, Prince M, Brayne C, Brodaty H, Fratiglioni L, Ganguli M (2005). Global prevalence of dementia: a Delphi consensus study. Lancet.

[3] Zaccai J, McCracken C, Brayne C (2005). A systematic review of prevalence and incidence studies of dementia with Lewy bodies. Age Ageing.

[4] McKeith I (2004). Dementia with Lewy bodies. Dialogues Clin Neurosci.

[5] Albert MS, DeKosky ST, Dickson D, Dubois B, Feldman HH, Fox NC (2011). The diagnosis of mild cognitive impairment due to Alzheimer’s disease: recommendations from the National Institute on Aging-Alzheimer’s Association workgroups on diagnostic guidelines for Alzheimer’s disease. Alzheimers Dement.

[6] Dubois B, Feldman HH, Jacova C, Cummings JL, Dekosky ST, Barberger-Gateau P (2010). Revising the definition of Alzheimer’s disease: a new lexicon. Lancet Neurol.

[7] Chételat G, Arbizu J, Barthel H, Garibotto V, Law I, Morbelli S (2020). Amyloid-PET and 18F-FDG-PET in the diagnostic investigation of Alzheimer’s disease and other dementias. Lancet Neurol Elsevier.

[8] Nobili F, Arbizu J, Bouwman F, Drzezga A, Agosta F, Nestor P (2018). European Association of Nuclear Medicine and European Academy of Neurology recommendations for the use of brain 18F-fluorodeoxyglucose positron emission tomography in neurodegenerative cognitive impairment and dementia: Delphi consensus. Eur J Neurol.

[9] Ramzan F, Khan MUG, Rehmat A, Iqbal S, Saba T, Rehman A (2019). A deep learning approach for automated diagnosis and multi-class classification of Alzheimer’s disease stages using resting-state fMRI and residual neural networks. J Med Syst.

[10] Ding Y, Sohn J, Mg K, H T, R H, Nw J (2018). A deep learning model to predict a diagnosis of Alzheimer disease by using ^18^F-FDG PET of the brain. Radiology.

[11] Choi H, Kim YK, Yoon EJ, Lee J-Y, Lee DS, Alzheimer’s disease neuroimaging initiative (2020). Cognitive signature of brain FDG PET based on deep learning: domain transfer from Alzheimer’s disease to Parkinson’s disease. Eur J Nucl Med Mol Imaging..

[12] Katako A, Shelton P, Goertzen AL, Levin D, Bybel B, Aljuaid M (2018). Machine learning identified an Alzheimer’s disease-related FDG-PET pattern which is also expressed in Lewy body dementia and Parkinson’s disease dementia. Sci Rep.

[13] Al-Shoukry S, Rassem TH, Makbol NM (2020). Alzheimer’s diseases detection by using deep learning algorithms: a mini-review. IEEE Access.

[14] Huang Y, Xu J, Zhou Y, Tong T, Zhuang X, Initiative (ADNI) the ADN. Diagnosis of Alzheimer’s Disease via Multi-Modality 3D Convolutional Neural Network. Front Neurosci [Internet]. Frontiers; 2019 [cited 2021 Apr 6];13. Available from: https://www.frontiersin.org/articles/10.3389/fnins.2019.00509/full. Accessed Jan-March 2021. 10.3389/fnins.2019.00509 PMC6555226 31213967

[15] Mehmood A, Maqsood M, Bashir M, Shuyuan Y (2020). A Deep Siamese Convolution Neural Network for Multi-Class Classification of Alzheimer Disease. Brain Sci. Multidisciplinary Digital Publishing Institute.

[16] Varrone A, Asenbaum S, Vander Borght T, Booij J, Nobili F, Någren K (2009). EANM procedure guidelines for PET brain imaging using [18F]FDG, version 2. Eur J Nucl Med Mol Imaging.

[17] Kramberger MG, Auestad B, Garcia-Ptacek S, Abdelnour C, Olmo JG, Walker Z (2017). Long-term cognitive decline in dementia with Lewy bodies in a large multicenter, international cohort. J Alzheimers Dis.

[18] McKeith IG, Dickson DW, Lowe J, Emre M, O’Brien JT, Feldman H (2005). Diagnosis and management of dementia with Lewy bodies: third report of the DLB Consortium. Neurology.

[19] Mueller SG, Weiner MW, Thal LJ, Petersen RC, Jack C, Jagust W (2005). The Alzheimer’s disease neuroimaging initiative. Neuroimaging Clinics of North America.

[20] Mazziotta JC, Toga AW, Evans A, Fox P, Lancaster J (1995). A probabilistic atlas of the human brain: theory and rationale for its development .The International Consortium for Brain Mapping (ICBM). Neuroimage.

[21] Simonyan K, Zisserman A. Very deep convolutional networks for large-scale image recognition. arXiv:14091556 [cs] [Internet]. 2015 [cited 2021 Apr 6]; Available from: http://arxiv.org/abs/1409.1556. Accessed Jan-March 2021.

[22] Zeiler MD, Fergus R, Fleet D, Pajdla T, Schiele B, Tuytelaars T (2014). Visualizing and understanding convolutional networks. Computer Vision – ECCV 2014.

[23] McInnes L, Healy J, Melville J. UMAP: uniform manifold approximation and projection for dimension reduction. arXiv:180203426 [cs, stat] [Internet]. 2020 [cited 2021 Apr 6]; Available from: http://arxiv.org/abs/1802.03426. Accessed Jan-March 2021.

[24] Fleiss JL (1971). Measuring nominal scale agreement among many raters. Psychol Bull US: American Psychological Association.

[25] Nichols TR, Wisner PM, Cripe G, Gulabchand L (2010). Putting the kappa statistic to use. Qual Assur J.

[26] Drzezga A, Grimmer T, Riemenschneider M, Lautenschlager N, Siebner H, Alexopoulus P (2005). Prediction of individual clinical outcome in MCI by means of genetic assessment and (18)F-FDG PET. J Nucl Med.

[27] Liu M, Cheng D, Yan W, Initiative ADN. Classification of Alzheimer’s disease by combination of convolutional and recurrent neural networks using FDG-PET images. Front Neuroinform [Internet]. Frontiers; 2018 [cited 2021 Apr 6];12. Available from: https://www.frontiersin.org/articles/10.3389/fninf.2018.00035/full. Accessed Jan-March 2021. 10.3389/fninf.2018.00035 PMC6018166 29970996

[28] Shen T, Jiang J, Lu J, Wang M, Zuo C, Yu Z, et al. Predicting Alzheimer disease from mild cognitive impairment with a deep belief network based on 18F-FDG-PET images. Mol Imaging. SAGE Publications Inc; 2019;18:1536012119877285. 10.1177/1536012119877285 PMC6764042 31552787

[29] Brown RK, Bohnen NI, Wong KK, Minoshima S, Frey KA (2014). Brain PET in suspected dementia: patterns of altered FDG metabolism. Radiographics.

